# DepressionMIGNN: A Multiple-Instance Learning-Based Depression Detection Model with Graph Neural Networks

**DOI:** 10.3390/s25144520

**Published:** 2025-07-21

**Authors:** Shiwen Zhao, Yunze Zhang, Yikai Su, Kaifeng Su, Jiemin Liu, Tao Wang, Shiqi Yu

**Affiliations:** 1 HACI Laboratory, Sydney Smart Technology College, Northeastern University, Shenyang 110167, China; 2472505@stu.neu.edu.cn (S.Z.); 2402087@stu.neu.edu.cn (Y.Z.); 2372307@stu.neu.edu.cn (Y.S.); 2372306@stu.neu.edu.cn (K.S.); 2 School of Computer Science and Engineeing, Northeastern University, Shenyang 110167, China; 3 Cloudlore Big Data Technology (Qinhuangdao) Co., Ltd., Qinhuangdao 066600, China; 18903340011@189.cn; 4 Daikin Comfort Technologies, Waller, TX 77484, USA; shiqi.yu@daikincomfort.com

**Keywords:** depression recognition, multimodality, multi-dimensional edge, graph neural networks

## Abstract

The global prevalence of depression necessitates the application of technological solutions, particularly sensor-based systems, to augment scarce resources for early diagnostic purposes. In this study, we use benchmark datasets that contain multimodal data including video, audio, and transcribed text. To address depression detection as a chronic long-term disorder reflected by temporal behavioral patterns, we propose a novel framework that segments videos into utterance-level instances using GRU for contextual representation, and then constructs graphs where utterance embeddings serve as nodes connected through dual relationships capturing both chronological development and intermittent relevant information. Graph neural networks are employed to learn multi-dimensional edge relationships and align multimodal representations across different temporal dependencies. Our approach achieves superior performance with an MAE of 5.25 and RMSE of 6.75 on AVEC2014, and CCC of 0.554 and RMSE of 4.61 on AVEC2019, demonstrating significant improvements over existing methods that focus primarily on momentary expressions.

## 1. Introduction

Depression, which is also referred to as depressive disorder [1], is now a common and serious mental disorder disease around the world. However, there is a shortage of mental health workers in most low- and middle-income countries. To aid mental health diagnosis and provide patients with early diagnosis, automatic depression estimation (ADE) technologies have been widely explored in recent years [2].

With the development of deep learning in affective computing [3,4,5,6], researchers have explored how to construct multimodal-based ADE systems. Some works have focused on feature fusion strategies to combine information from different modalities like visual, audio, and text. Various fusion methods have been proposed such as equal-weighted fusion or attention-based fusion [7,8,9]. Other works have developed multimodal neural network architectures [10] including CNNs [11], RNNs, and transformers tailored [12] for each modality before fusing them. Some researchers have specifically focused on capturing temporal dependencies by employing multi-scale temporal CNNs and transformers to model long-range dependencies [13,14,15]. However, most existing approaches still focus on identifying transient moments in a video that represent depression symptoms. This setting hypothesizes that depression is a temporary expression rather than a persistent state.

**Figure 1:**
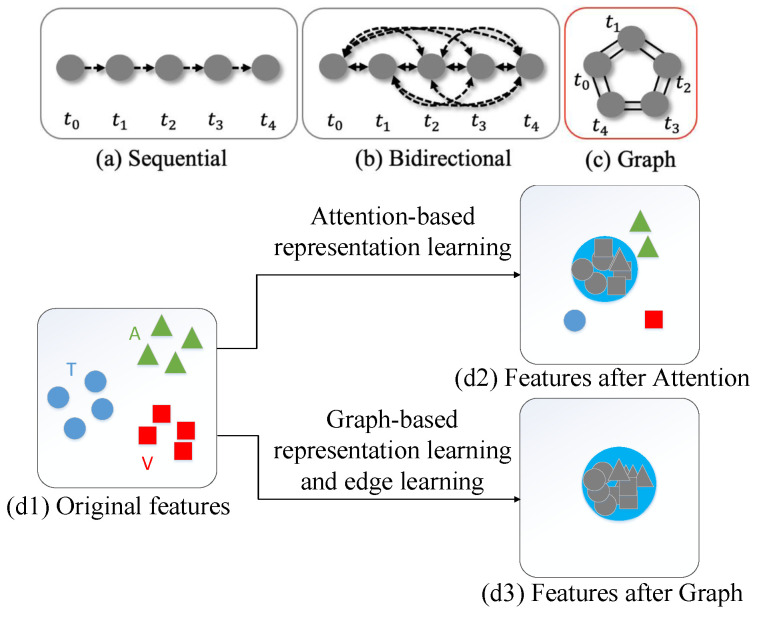
Differences in temporal connection type between existing methods and our method, a different state of features: (**a**) sequential connection; (**b**) bidirectional full connection; (**c**) the proposed graph-based connection with a multi-dimensional edge; (**d**) hidden features, where different colors represent different subintervals; (**d1**) is the original three modal features, which are not aligned; (**d2**) is the feature after being aligned by the multi-attention mechanism, with many outliers; (**d3**) is the feature after being aligned by the graph network—all are aligned to the same subspace.

**Limitations of existing methods:** Despite the progress made in multimodal ADE systems, existing methods suffer from three major limitations. First, as illustrated in Figure 1a,b, current temporal modeling approaches primarily utilize GRU to analyze sequential connections or employ attention mechanisms to explore bidirectional full connections of temporal information. However, the sequential connection approach fails to account for jump-connected event relationships [16], while the bidirectional full connection approach, though aiming to capture key segments, disrupts the chronological logic of temporal event development to some extent.

Second, according to clinical descriptions of depression symptoms [17,18,19], depression is fundamentally a chronic disorder that progressively worsens over time, manifesting as a persistent state rather than momentary expressions even within a single interaction episode. Current approaches that focus on identifying transient depressive moments fail to capture this essential characteristic of depression as a sustained condition that requires analysis of feature-aware past–future chronological development and associations among intermittent relevant events.

Third, as illustrated in Figure 1(d1), multimodal features from audio, text, and video modalities (represented by different colors for different subspaces) are naturally unaligned in high-dimensional space. Existing alignment and fusion methods, namely, direct concatenation and multi-head attention mechanisms, have significant limitations. Direct concatenation simply connects feature vectors sequentially, ignoring dynamic correlations and temporal dependencies between modalities, resulting in shallow and context-unaware fusion. While multi-head attention mechanisms consider contextual associations, they typically learn all relationships from scratch to capture temporal and crossmodal associations, making them sensitive to data and feature quality and potentially suboptimal. As shown in Figure 1(d2), these approaches lead to incomplete modal alignment and suboptimal feature fusion.

**Proposed solution:** To address these limitations, this paper proposes a novel graph-based temporal depression representation alignment and learning approach. Our method addresses the first limitation by designing two-dimensional edges (Figure 1c)—forward-full-connection (FFC) and backward-full-connection (BFC)—to construct graphs with multimodal utterance embeddings, enabling consideration of both past–future chronological development and associations among intermittent relevant events. For the second limitation, our approach segments multimodal long-term samples into utterance-level instances processed with bidirectional GRU, maintaining the integrity of chronological development logic while capturing the chronic nature of depression symptoms. To solve the third limitation, we employ relational graph convolutional neural networks (RGCN) [20] and Graph Attention Networks (GATs) [21] for dynamic learning of multimodal features. Through learning to aggregate nodes from different modalities, our graph network effectively aligns and fuses features at the feature level, as demonstrated in Figure 1(d3), successfully aligning all three modalities to the same subspace.

The main contributions to address the discussed three problems of this paper can be summarized as follows:

- We segment the multimodal long-term sample into multiple instances at the utterance level and process them with the bidirectional GRU, so that the segment-level contextual information can be well captured.
- We design two-dimensional edges (see Figure 1c, forward-full-connection (FFC) and backward-full-connection (BFC), to construct a graph with multimodal utterance embeddings, so that the past–future chronological development and the association among intermittent relevant information can be considered.
- We propose a novel graph-based temporal depression representation alignment and learning approach with a relational graph convolutional neural network (RGCN) [20] and Graph Attention Network (GAT) [21] to analyze the multimodal features by making full use of the two relation types. We use the graph network for dynamic learning of multimodal features, through learning to aggregate the nodes from different modalities, and then effectively align and fuse them at the feature level, as shown in Figure 1(d3); finally, the three modalities are effectively aligned to the same subspace.

The remainder of this paper is organized as follows. In Section 3, the architecture we propose is presented with a focus on graph network-based multimodal fusion techniques. In Section 4, the experimental preparation and experimental setup are described, as well as the results with an in-depth discussion and analysis. Additionally, Section 2 and Section 5 provide an introduction to related works and a summary of this paper, respectively.

## 2. Related Works

Multimodal data processing encompasses various fusion techniques, including early fusion, late fusion, and model-based fusion. Early fusion, also referred to as feature-level fusion, entails integrating data features from disparate modalities at the initial stage of data processing, thereby facilitating an in-depth exploration of crossmodal interactions and correlations. However, this approach is constrained by intermodal mismatches and the complexity of high-dimensional data. Late fusion, which involves fusing results after each piece of modal data is processed independently, is more suitable when modalities exhibit greater independence and simplifies processing, but may overlook potential correlations between modalities. Model-based fusion, on the other hand, performs fusion directly within the model through learning algorithms, such as graph networks or integrated learning strategies, which can adapt flexibly to the characteristics of different modalities, but necessitate more stringent algorithm design requirements. Notably, early and late fusion techniques often employ attention mechanisms or direct feature splicing, while model-based fusion frequently utilizes graph-based representation learning and edge learning.

### 2.1. Fusion Based on Feature Splicing

Among the feature fusion strategies, the fusion method based on dimensional splicing is a commonly used and effective strategy. This method realizes the fusion of multi-source information by splicing features from different data sources or feature extraction methods in feature dimensions. Through dimensional splicing, features of different types or dimensions can be combined together to expand the feature space and improve the model’s representation of data. In [22], they proposed a set of multimodal and multiresolution feature extraction methods for detecting depression through speech and facial marker features, and explored the model performance corresponding to the fusion strategy of audio and video features at early and late stages, respectively. In recent years, deep neural networks have been developing rapidly, so more and more neural network-based multimodal fusion methods are being used to better accomplish upstream tasks. To address the challenges of cross-language and cross-cultural depression prediction. In [7], they proposed a GRU-based trimodal fusion network model for text, video, and audio, which effectively captures complex signals related to depression severity and cross-cultural sentiment recognition, demonstrating the potential and effectiveness of multimodal analysis in the field of sentiment computing. In the same year, ref. [10] also extracted and fused the features of the three modalities using BERT-CNN, VGG-GCNN, and ResNet-50, respectively, and the experimental results showed that the extraction of the features had a large impact on the model performance. To effectively fuse modalities and simultaneously investigate the impact of global topic information of text and images on the depression detection task, they first proposed a new Multimodal Topic Augmented Assisted Learning (MTAL) method, which aims to capture topic information within different modalities to enhance depression detection, by proposing a modality-independent topic model capable of mining topic cues from discrete textual signals or continuous visual signals to assist in depression detection tasks [23]. In [24], they perform feature extraction on text and speech separately by analyzing the features in speech and text, respectively, applying an attention mechanism to the text to highlight depression-related elements, and finally feature stitching for depression detection.

### 2.2. Fusion Based on Attention Mechanisms

In multimodal feature fusion, the attention mechanism plays a crucial role in emphasizing the information most critical for diagnosis by assigning different weights to different features of each modality. For example, when fusing speech, video, and text data, the attention model can learn that subtle changes in facial micro-expressions, which are more indicative of a patient’s depressive state than the tone of voice, and thus prioritize this part of the data. Recent advances in attention-based multimodal fusion have demonstrated significant improvements across various domains. The effectiveness of attention mechanisms in multimodal tasks has been widely validated, with studies showing that enhanced attention networks can effectively integrate information from multiple views or modalities [25]. These findings underscore the importance of sophisticated attention mechanisms for complex multimodal analysis tasks, including depression detection where different modalities may contribute varying levels of diagnostic information. To address the problem of efficiently extracting depression-related cues from speech and facial activities, ref. [26] proposed an approach that combines a spatial–temporal attention network (STA) and a multimodal attention feature fusion strategy (MAFF) by segmenting speech and video data and using the attention mechanism to emphasize the depression detection-related features, finally generating multimodal representations with modal complementary information. In order to fully explore the impact of semantic content and visual information on depression assessment, they proposed that the application of the deep learning model Bi-GRU combined with the attention mechanism can effectively recognize depression. Meanwhile, the attention mechanism is utilized to enhance the association learning between visual and textual modalities as a way to improve the accuracy and efficiency of depression detection [27]. To better extract depression features and fuse audio and text features, they used a combination of GRU and BiLSTM to deeply analyze the mood fluctuations in the audio and the semantic information in the text, so as to effectively recognize depression. Finally, the attention mechanism was applied to the two modalities to effectively fuse the features [28]. In order to improve the accuracy of sentiment analysis and depression detection, ref. [13] established a tensor-based multimodal transformer model, TensorFormer, which, through its global cross-attention module and parallel feed-forward module, allows information from different modalities to be comprehensively interacted and fused, thus improving the performance and flexibility of the model when processing multimodal data. In [29], they proposed a network based on contextual attention and information interaction mechanisms that can capture important acoustic and visual features at critical time points and extract correlations and interactions between acoustic and visual features at local and global scales.

### 2.3. Fusion Based on Graph Network

In practical applications, numerous types of information can be constructed into non-grid topologies, such as social networks [30] and character interactions [31], and traditional convolutional neural networks face challenges in processing such non-Euclidean data. Therefore, ref. [32] firstly proposed the Graph Neural Network (GNN), which can directly process the graph structure. However, the classical GNN has limitations, such as using the same parameters in the iteration, and it is difficult for the model to learn deeper feature expressions. To address this, ref. [20] proposed transferring information from neighboring nodes to the target node to perform graph convolution operations, which was the first time the convolution operation in image processing was simply used in graph structure data processing. Nevertheless, Graph Convolutional Networks (GCNs) assign the same weight to different neighbors in the neighborhood of the same order, limiting the ability of the GCN model to capture the relevance of spatial information. Recently, the attention mechanism has received increasing attention from scholars, so [21] proposed using the attention mechanism for weighted summation of neighboring node features, where the weights of neighboring node features depend entirely on the node features and are independent of the graph structure. Due to the rapid development of graph networks in prediction tasks, scholars have applied them to the field of disease detection. When using multimodality for graph embedding for depression detection, ref. [33] proposed a novel hierarchical context-aware graph attention model for automated depression detection, simulating the hierarchical structure of depression assessment and using a graph attention network to capture relational contextual information in text and audio modalities. To model the dynamic fusion between modalities, ref. [34] proposed a multi-head intermodal attention mechanism based on GAT. By utilizing the powerful ability of graph attention networks to capture complex dynamic relationships between different modalities, and by using multi-head attention, the model can focus on different subsets of information simultaneously, enhancing the model’s ability for multimodal data fusion. This intermodal attention mechanism enables the model to better understand and integrate information from different modalities, such as speech, text, and visual signals. To explore the heterogeneity/homogeneity among various modalities, ref. [35] proposed a multimodal fusion method called MS2-GNN, which can explore the heterogeneity/homogeneity among multiple physiological and psychological modalities and study the differential relationships among individuals. The modality-shared GNN and modality-specific GNN architectures are utilized to extract intermodal/intramodal features, respectively. However, it does not consider that depression is a chronic long-term illness and does not model the entire session data temporally, nor does it model the node embedding of individual sessions using graph networks, and it does not perform the analysis of jump–connection event relationships.

To capture long-term temporal patterns of depression and achieve effective multimodal fusion, we segment data into discourse-level instances and obtain contextual representations via GRU. These are embedded as nodes in a graph, where RGCN and GAT are used to model intermodal relationships and temporal dependencies. As summarized in Table 1, prior studies mainly rely on handcrafted fusion or attention-based methods, often overlooking the chronic and sequential nature of depression. Our approach addresses this gap by introducing dual-edge temporal graph modeling, enabling fine-grained alignment and session-level behavioral reasoning.

## 3. Materials and Methods

The proposed model introduces a new approach to analyzing long-term and episodic temporal dependencies for depression. The experimental data are sourced from established multimodal datasets, which include synchronized video, audio, and text streams. These modalities are collected using sensors such as RGB cameras and condenser microphones—commonly embedded in consumer-grade devices like webcams and smartphones—under controlled recording environments. The video recordings provide facial expressions and head movements, while the audio captures vocal tone, rhythm, and energy. These sensor-acquired signals offer crucial cues for assessing mental health status.Unlike previous approaches that mainly rely on momentary or handcrafted fusion mechanisms, our framework is tailored to depression’s long-term and episodic nature by learning temporal dependencies through a graph-based formulation.

First, video is segmented into utterance-level instances and encoded into contextual representations, so as to analyze short-term features. These contextual representations are processed as nodes in a graph with dual connections to model both chronological development and relevant intermittent information among nodes. This allows the model to capture multi-dimensional temporal dependencies that are critical for understanding depression disorders.

### 3.1. Preprocessing

The multimodal data used in this study were originally collected through commonly used sensor devices. Specifically, visual signals were captured using RGB cameras at a frame rate of 30 frames per second (FPS), and audio signals were recorded through standard microphones at a sample rate of 16 kHz. These sensors recorded participants’ facial expressions, vocal characteristics, and spoken content under controlled interview or conversational settings. The transcribed text was obtained by applying automatic speech recognition (ASR) to the audio recordings. To clarify the segmentation by utterance using ASR, we directly utilize the transcriptions provided by the AVEC2019 dataset, which were pre-processed by the dataset creators using automatic speech recognition. The dataset provides utterance-level transcriptions with corresponding timestamps. We simply extract features from each pre-defined utterance segment: audio features from the temporal boundaries specified in the dataset, visual features from the corresponding video frames, and textual features from the provided transcribed content. This approach ensures consistent temporal alignment across all three modalities based on the dataset’s established utterance boundaries.

Assuming that we have a dataset $D={\left\{S_{id},Y_{id}\right\}}_{id=1}^{N_{s}}$, $S_{id}$ is the sample of a subject that consists with $\left[X_{id}^{A},X_{id}^{V},X_{id}^{T}\right]$. $X_{id}^{A}$ represents the acoustic features extracted from speech, $X_{id}^{V}$ represents the visual features extracted from facial expressions, and $X_{id}^{T}$ represents the linguistic features extracted from speech transcripts. $Y_{id}$ is the corresponding BDI or PHQ-8 score. BDI (Beck Depression Inventory) is a 21-item self-report measure designed to assess depressive symptom severity. PHQ-8 (Patient Health Questionnaire-8) is a clinically validated 8-item scale widely adopted for screening and grading depression severity. The proposed approach considers information from the audio modality, visual modality, and text modality. In order to ensure that all three modalities are available at the same time and aligned with each other, we intercepted samples according to the moment of the speaker’s utterances and re-organize them in the temporal order.

**Acoustic features**: Acoustic features were extracted using the OpenSmile toolkit with IS10 configuration [36]; $X_{id}^{A}=\left[x_{0}^{a},x_{1}^{a},\ldots ,x_{n}^{a}\right]$, where id is the index of the sample and *n* is the number of utterances. The same notation applies below. We divided each speech recording using a sliding window of 4 s with a 1-second step size, and extracted feature vectors within each window using the Bag-of-audio-words eGeMAPS [37] approach. The 100-dimensional feature vector is empirically determined based on the Bag-of-audio-words approach, which is commonly used in the audio processing literature for effective acoustic representation. Finally, the dimension of $X_{id}^{A}$ is (n,100).

**Visual features**: Facial features were extracted from video clips using a DenseNet pretrained on the FER+ dataset [38] as $X_{id}^{V}=\left[x_{0}^{v},x_{1}^{v},\ldots ,x_{n}^{v}\right]$. The DenseNet was pretrained on the FER+ dataset for classifying eight basic emotions using cross-entropy loss. We first extract the region of facial expression and align it with the OpenFace toolkit, and then feed the aligned face images to the pretrained ResNet-50 [39] to obtain the deep representation. The 2048-dimensional vector follows the standard output dimension of ResNet-50’s feature layer, which is widely adopted for visual feature representation in multimodal analysis.

**Textual features**: We utilized a fine-tuned RoBERTa Large model [40] for text transcripts, appending an <S> token to tokenized utterances as $X_{id}^{T}=\left[x_{0}^{t},x_{1}^{t},\ldots ,x_{n}^{t}\right]$. We employed a pretrained BERT model to convert the transcript into sentence embeddings. The 768-dimensional representation corresponds to the hidden state size of the BERT model, which is the standard dimension for BERT-based text embeddings. The dimension of textural features is (n,768).

### 3.2. Model Architecture

**Figure 2:**
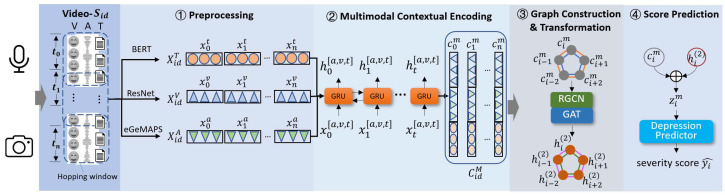
Architecture of the proposed model. Firstly, the input video sample, collected via sensors, is divided into multiple instances at the utterance-level. The BoAW eGeMAPS, pretrained ResNet, and pretrained BERT models are utilized to extract audio, visual, and textual features. Bidirectional GRUs are then used to update the multimodal features across time-steps. The graph is constructed initially with the designed FFC and BFC. RGCN and GAT models are employed to update the node features and connections. Finally, the updated node features are concatenated with contextual representations and used for PHQ score prediction.

The proposed DepressionMIGNN has 3 procedures as depicted in Figure 2: (1) Multimodal Context Encoding, (2) Graph Construction and Transformation, and (3) Score Prediction.

**Multimodal Context Encoding:** As the features obtained from the pre-processing procedure only reflect short-term information, to account for contextual information, inspired by [41], bidirectional GRUs were utilized to update features across time-steps. The following equations adapted from [42] demonstrate the process.

(1) $$c_{i}^{\left[a,v,t\right]},h_{i}^{\left[a,v,t\right]}=\overset{↔}{GRU}\left(x_{i}^{\left[a,v,t\right]},h_{i-1}^{\left[a,v,t\right]}\right)$$

where $\overset{↔}{\mathrm{GRU}}\left(\cdot \right)$, indicates that the outputs of the forward GRU and the backward GRU are concatenated along the channel dimension. Here, $h_{i}^{\left[a,v,t\right]}$ represents the updated hidden state for each modality, and $c_{i}^{\left[a,v,t\right]}$ denotes the context-enhanced representation obtained by concatenating forward and backward outputs, which is then used for subsequent multimodal fusion. Each modality is processed with an independent GRU, and their weights are not shared. After the contextual representation is obtained for each modality, the representations of the three modalities are concatenated to jointly represent a short-term segmentation:

(2) $$c_{i}^{m}=c_{i}^{a}⊕c_{i}^{v}⊕c_{i}^{t};\;\;C_{i}=\left[c_{0}^{m},c_{1}^{m},\ldots ,c_{n}^{m}\right]$$

where $C_{i}$ denotes the multimodal context matrix of the *i*-th short-term segment.

**Graph Construction and Feature Transformation:** Given the multimodal contextual representation, we construct a temporal graph G=(V,E,R,W) where each node represents a segment (utterance) from the sample. Specifically, each node $v_{i}\in V$ corresponds to the multimodal feature representation $c_{i}^{m}$ of an utterance segment with dimension 160. V refers to the set of nodes (utterance segments), E denotes the set of edges connecting these segments, R represents the types of temporal relationships among consecutive and non-consecutive utterances (including forward and backward temporal connections), and W contains the corresponding edge weights that are learned and optimized during training. The edges $e_{ij}\in E$ are generated based on temporal proximity and semantic similarity between utterance segments $c_{i}^{m}$ and $c_{j}^{m}$. Each edge $e_{ij}$ has a relation type r∈R and a weight $\alpha_{ij}\in W$ with $0\leq \alpha_{ij}\leq 1$, which is updated through the training process.

Additionally, a window range $N_{w}$ is implemented around a central utterance when constructing the graph to constrain the number of nodes. This aims to improve the model’s ability to capture relationships within a specific time period. In detail, we selected $\frac{N_{w}}{2}$ sentences before and after the current central utterance to form the graph nodes, meaning that $\frac{N_{w}}{2}$ refers to the number of sentences on each side of the central one. The graph hops along the time sequence with a step length of 1.

As Figure 2 demonstrates, we designed two relation types, FFC and BFC. The adjacent matrices are learnable with an attention-like process as Equation (3) shows.

(3) $$\alpha_{ij}=\mathrm{softmax}\left(c_{i}^{mT}W_{e}^{r}\left[c_{i-\frac{N_{w}}{2}}^{m},\ldots ,c_{i+\frac{N_{w}}{2}}^{m}\right]\right)$$

where $W_{e}^{r}$ is a trainable weight matrix for the relation type *r*. Once the graph is constructed, we update the features with graph neural networks. First, we use RGCN to update the node representation according to our definition of both FFC and BFC connections, considering the past-future chronological development.

(4) $$h_{i}^{\left(1\right)}=\sigma \left(\sum_{r\in R}\sum_{j\in N_{i}^{r}}\frac{\alpha_{ij}}{N_{i}^{r}}W_{r}^{\left(1\right)}c_{j}^{m}+\alpha_{ii}W_{0}^{\left(1\right)}c_{i}^{m}\right)$$

where $W_{r}^{\left(1\right)}$ and $W_{0}^{\left(1\right)}$ refer to weight matrices that are learned during training. The variable α represents the weights of the edges, and $N_{i}^{r}$ is the set of indices for nodes in the neighborhood of node *i* under the relation *r*. The activation function is denoted by σ. Next, in order to capture the association among intermittent relevant information, we utilized GAT with the RGCN output features $h_{i}^{1}$ as input to compute the new connection weight $\alpha^{\prime }$ based on the updated features, followed by another round of feature updating with the new weights, as shown in Equation (5), which is derived from the paper on Graph Attention Networks cited in [21]:

(5) $$\begin{array}{cc} \alpha_{ij}^{\prime } & =\frac{exp(a^{T}LeakyReLU\left(W_{g}^{\left(2\right)}\left[h_{i}^{\left(1\right)}⊕h_{j}^{\left(1\right)}\right]\right))}{\sum_{j\in N_{i}^{r}}exp\left(a^{T}LeakyReLU\left(W_{g}^{\left(2\right)}\left[h_{i}^{\left(1\right)}⊕h_{j}^{\left(1\right)}\right]\right)\right)}\\ h_{i}^{\left(2\right)} & =\alpha_{ii}W_{g}^{\left(2\right)}h_{i}^{\left(2\right)}+\sum_{j\in N_{i}^{r}}\alpha_{ij}W_{g}^{\left(2\right)}h_{j}^{\left(2\right)} \end{array}$$

where $W_{g}^{\left(2\right)}$ represents a learnable weight matrix, a represents a parameterized weight vector, and LeakyReLU is an activation function.

To stabilize the learning process of self-attention, we found that extending our mechanism to employ multi-head attention was beneficial. Specifically, *K* independent attention mechanisms execute the transformation of Equation (6), and then their features are concatenated, resulting in the following output feature representation:

(6) $$h_{i}^{\left(2\right)}={\|}_{k=1}^{K}\left(\alpha_{ii}^{k}W_{g}^{(2),k}h_{i}^{\left(2\right)}+\sum_{j\in N_{i}^{r}}\alpha_{ij}^{k}W_{g}^{(2),k}h_{j}^{\left(2\right)}\right)$$

where ‖ represents concatenation, $\alpha^{k}$ are normalized attention coefficients computed by the k-th attention mechanism, and $W_{g}^{\left(2\right)}$ is the corresponding input linear transformation’s weight matrix.

**Subject-Level Prediction:** It is important to clarify that the AVEC datasets used in our study provide depression severity scores at the subject level, not at the utterance level. Therefore, all utterances from a given subject share the same label. Following the Multiple-Instance Learning (MIL) paradigm, we segment each video into utterance-level instances to allow the model to capture fine-grained temporal patterns within the session. These utterances are treated as instances in a bag, and the model is trained to make predictions based on the collective evidence of all utterances.

The MIL formulation offers several advantages for depression detection: it enables automatic identification of the most diagnostically relevant utterances rather than treating all segments equally, handles noise in long-term behavioral data by focusing on discriminative instances, and aligns with clinical assessment practices where depression is evaluated based on overall symptom patterns. The subject-level prediction is obtained by averaging the instance-level predictions from all utterances, ensuring the final score reflects collective evidence while allowing the model to emphasize more informative temporal segments through learned representations. This approach enables our framework to focus on the most informative segments while still producing a subject-level prediction, aligning with the nature of depression as a long-term disorder.

**Score Prediction:** To predict the BDI/PHQ-8 score, we first concatenate the contextual representations $c_{i}^{m}$ and node features $h_{i}^{\left(2\right)}$. Then, we perform predictions for each utterance-level short-term information and calculate the average score of all utterances as the final estimation result. The PHQ-8 and BDI scores are continuous values that quantify depression severity, where higher scores indicate more severe depressive symptoms. These scores are provided as ground truth labels in the AVEC dataset at the subject level. Our model outputs a regression score that directly corresponds to these clinical assessment scales, enabling clinicians to interpret the results in terms of established depression severity categories.

(7) $${\hat{y}}_{i}=\sigma \left(W_{c}\left[c_{i}^{m}⊕h_{i}^{\left(2\right)}\right]+b_{c}\right);\;\;{\hat{Y}}_{id}=\frac{\sum_{i\in n}{\hat{y}}_{i}}{n}$$

where σ denotes the activation function, $y_{i}$ is the predicted score for utterance *i*, $W_{c}$ is a learnable weight matrix, and $b_{c}$ is bias.

We adopted Concordance Correlation Coefficient (CCC) loss as the cost function during the training procedure.

(8) $$CCC=\frac{2\rho \sigma_{f}\sigma_{y}}{\sigma_{f}^{2}+\sigma_{y}^{2}+{\left(\mu_{f}-\mu_{y}\right)}^{2}}\;,\;L_{CCC}=1-CCC$$

where ρ is the Pearson correlation coefficient, $\mu_{f}$ and $\mu_{y}$ are the mean values of predictions and ground truth labels, respectively, and $\sigma_{f}$ and $\sigma_{y}$ are the corresponding standard deviations. The value of CCC ranges from −1 to 1, where 1 denotes an ideal positive correlation and −1 denotes a completely negative correlation.

## 4. Experiment

In this section, we introduce the public benchmark dataset, the baseline methodology used for comparisons, as well as the detailed experimental results and visualizations.

### 4.1. Dataset and Baselines

We conduct experiments on two widely used multimodal depression datasets: **AVEC2014** and **AVEC2019**, summarized in Table 2. AVEC2014 includes audio–visual data from 82 subjects across two tasks (Northwind and Freeform), with depression levels labeled using the BDI. The two tasks were combined for the experiments and equally divided into the training set, development set, and test set, resulting in 100 video samples for each set. Each set therefore includes approximately 100–200 min of video data in total, depending on the response duration of each subject. And the average duration for each sample is 1 to 2 min. AVEC2019 is built upon the E-DAIC corpus and contains data from 275 subjects, where the depression level is annotated using the PHQ-8. In AVEC2019, the transcripts were derived from semi-automatic transcriptions with manual correction. The entire AVEC2019 corpus contains approximately 73 h of semi-clinical interviews, with the training set covering about 43 h and the development and test sets each around 15 h.

To validate our model, we compare it with a series of baselines ranging from traditional machine learning regressors to recent deep multimodal models. These methods include handcrafted feature pipelines, attention-based fusion, temporal modeling, and transformer-based approaches. Table 3 provides a summary of representative baselines and their key modeling strategies.

### 4.2. Settings and Metrics

We develop DepressionMIGNN using PyTorch 1.6.0 and PyG 1.6.3 as frameworks. The dimensions of processed audio, visual, and textual features in the preprocessing procedure are 100, 2048, and 768, respectively. The number of cells in the GRU used in the Multimodal Contextual Encoding is 200, and the number of utterances per subject ($N_{w}$) is 20. The learning rate was set to $1\times 10^{-4}$ with a weight decay of $1\times 10^{-8}$. The model was trained for 500 epochs with a batch size of 50. Training was conducted on a single NVIDIA RTX 4070 GPU (12 GB), with a peak memory usage of approximately 5 GB. On average, each training epoch took around 12 s. Our proposed model takes approximately 100 min to train on the training set and around 20 min to evaluate on the test set.

To evaluate and compare the performance of our method with selected baselines, we utilized two commonly used metrics in previous studies: the Concordance Correlation Coefficient (CCC) and the Root Mean Square Error (RMSE).

### 4.3. Results

#### 4.3.1. Sensor Requirements and Modal Usage

To apply the proposed depression detection method in real-world settings, only two commonly available sensors are required: an RGB camera and a microphone. The RGB camera captures facial movements and visual cues, while the microphone records acoustic signals during spontaneous speech. These sensors are typically embedded in consumer devices such as laptops and smartphones, making the system hardware-efficient and highly deployable.

In our implementation, the recorded audio and video streams are temporally synchronized and segmented into utterances. Visual frames are aligned and processed using a pretrained ResNet-50 to extract facial features, while acoustic signals are analyzed using the eGeMAPS feature set via the OpenSmile toolkit. Transcripts are generated through automatic speech recognition (ASR), and semantic features are encoded using a pretrained RoBERTa model. These three modalities are fused and modeled through our graph-based architecture to capture both short- and long-term temporal patterns indicative of depressive symptoms.

#### 4.3.2. Comparison Experiment Results

Table 4 presents the results of DepressionMIGNN and baselines. The Parameters column indicates the number of model parameters. In the table, it can be seen that our proposed model outperforms the selected baselines on the CCC metric. To elucidate the reason for the improvement, we should examine the fundamental differences between our approach and the baselines. The baseline approaches, refs. [7,10,43,44,45,46,48,50], focus on fusing features without placing much emphasis on the relationship between temporal information. In the AVEC2014 dataset experiment, ref. [26] with 2.6 M parameters did slightly better than us on MAE, but we significantly improved on RMSE compared to this model. Although [8] with 4.2 M parameters used a multiscale dilated CNN to consider temporal information, this approach has limitations in handling temporal features and cannot consider long time intervals at once. And although [47] with 32.1 M parameters can capture the global and local spatial–temporal information, the parameters of its model are too large. Although [13] with 65 M parameters utilized the transformer’s ability to capture long time dependencies, the attention mechanism’s processing of temporal information aims to capture key segments and is somewhat disruptive, without considering the back-and-forth logic of temporal event development. In addition, ref. [53], while considering the effect of affective states on depression, did not adequately consider the contextual information and did not consider the prolonged dependency of depression during the interaction. These methods are insufficient in fully matching the characteristics of depressive symptoms, chronic, sporadic, and intermittent. Our FFC and BFC are designed to preserve the chronological development logic of depressive symptoms at the vide-level, which aligns with the chronic nature of the condition. Our graph-based method also utilizes the adjacency matrix to capture non-sequential connections between temporal events, which is in line with the sporadic and intermittent nature of depressive symptoms. This approach allows for a more comprehensive representation of the temporal dynamics of depression, which ultimately improves the accuracy of depression estimation.

As shown in Table 4, our proposed model achieves competitive performance across both datasets. On AVEC2014, we obtain the lowest RMSE (6.75) among all models and a near-best MAE (5.25), slightly higher than MAFF (5.21). On AVEC2019, our model achieves a CCC of 0.554, which is on par with the best-performing method FAU-GF (0.555) and outperforms most other baselines. Our RMSE (4.61) is also competitive, only slightly higher than TensorFormer (4.31), while using less than 1/10th of its parameters (6.1 M vs. 65 M).

These results demonstrate that our model achieves a strong trade-off between accuracy and computational efficiency. In particular, the high CCC scores validate the effectiveness of our temporal graph-based fusion in capturing long-term depressive patterns.

#### 4.3.3. Ablation Study

In order to investigate the amount of depression information contained in different modalities, an ablation study is conducted by comparing the performance of different combinations of modalities. In addition, we discuss the contribution of the components employed in the proposed model. Specifically, we removed multi-instance learning, GNN, GAT, and the designed relations one by one, and compare the performance of different model configurations.

Table 5 shows the performance of the unimodal and multimodal models on the AVEC2014 dataset and AVEC2019 dataset, where A denotes audio modality, V denotes visual modality, and T denotes text modality. As shown in Table 5, among the single modalities, the textual modality (T) achieves the best performance, highlighting the importance of semantic information in depression detection. Among bimodal combinations, T + A performs the best, indicating strong complementarity. V + A does not outperform unimodal A, suggesting limited contribution from visual features alone. The full trimodal configuration (T + A + V) achieves the best results overall, confirming the effectiveness of multimodal integration despite modality-specific noise.

Furthermore, we discuss the contribution of the components employed in the model. Specifically, we remove the multi-instance learning strategy, the GAT module, the GNN module, and the bidirectional relation to compare the performance advantages and disadvantages of the different models, and the results are also shown in Table 5. It can be found that the absence of any of these components leads to a sharp drop in performance.

The multi-instance learning strategy enhances the model’s understanding of time series data by segmenting video samples into multiple instances at the utterance level, combining them with an GRU model to extract rich contextual information, and effectively enhancing the model’s understanding of time series data. This enables the model to capture detailed information within each instance and broader temporal dependencies through contextual relationships between instances, thus improving the accurate prediction of depression representations. Without the multi-instance module, the model loses the ability to understand video content at a fine-grained level, resulting in a significant decrease in the ability to capture temporal dependencies and overall confidence.

The GNN module, particularly with bi-directional connectivity relationships, significantly enhances the model’s ability to fuse time series data, enabling the model to combine past and future information and more accurately reflect the complex associations between temporal development and intermittently relevant information. This approach demonstrates significant advantages in capturing subtle changes in depression video samples and is particularly important for understanding and predicting time-dependent manifestations of depression. We also found that introducing only GCN without invoking the GAT module, although improved relative to the model that does not use GNN at all, still performs very poorly relative to the model that uses both GCN and GAT together. This is because GCN updates the representation of each node mainly by averaging or summing the features of neighboring nodes, which, although capable of capturing graph structural information, is not enough to capture all important feature interactions and subtle differences between modalities. In contrast, GAT is able to capture relationships between nodes in a more fine-grained way by assigning different importance to different edges through the attention mechanism. Finally, we conducted ablation experiments on bi-directional connectivity relationships, considering only the sequential relationships of discourse, and found that the predictive performance of the model is unsatisfactory, leading to the loss of important contextual information and interaction patterns. Bidirectional connections help capture the interplay and support of discourse between users, reflecting the true complexity between utterances. When only unidirectional discourse order relationships are considered, the model is unable to fully understand the dynamic associations between user behaviors and mental states, leading to a reduced ability to identify the phenomenon of depression.

#### 4.3.4. Effect of Different Numbers of Windows

To investigate the optimal context window size for capturing sporadic depressive symptoms, we conducted experiments with varying window sizes. Using (10,10) as the baseline, we tested increasing window sizes of (12,12), (15,15), and (20,20), and decreasing window sizes of (8,8), (5,5), and (0,0). The results (Table 6) show that the model’s performance degrades gradually as the window size deviates from the optimal value of 20 utterances, suggesting that this interval is most indicative of depressive mood expression in this dataset. Notably, the model with a context window size of (0,0), equivalent to a sequential encoder-only model, performs significantly worse, highlighting the importance of contextual information.

#### 4.3.5. Effect of Different Numbers of Heads

**Figure 3:**
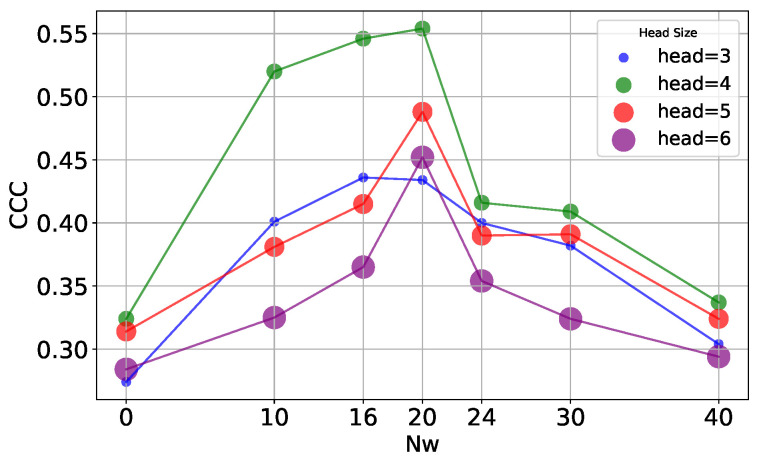
CCC results with the proposed method for different combinations on the AVEC2019 dataset, with different $N_{w}$ values on the horizontal axis and CCC values on the vertical axis, and different colors and bubble sizes in the graph for different numbers of heads.

**Figure 4:**
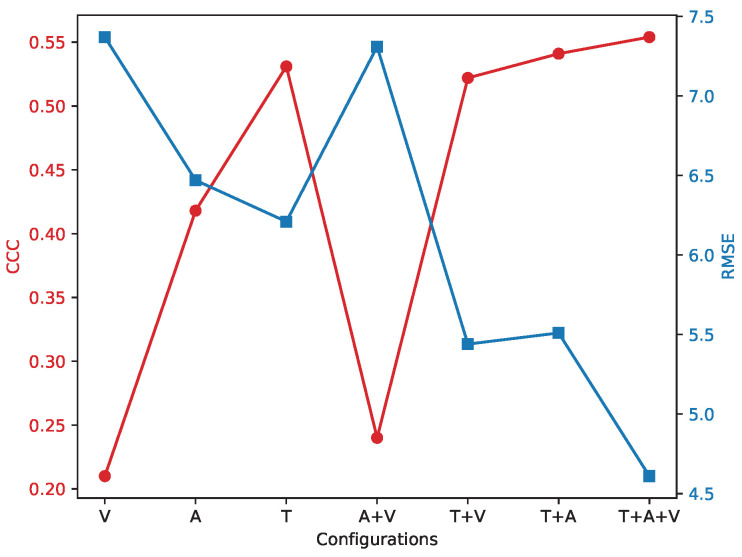
Plot of the experimental results of modal ablation on the AVEC2019 dataset when $N_{w}$ = 20, head = 4 with the proposed method, where the horizontal axis is the different modal ablation models, the red line represents the CCC index, and the blue curve represents the RMSE index.

Since this model uses the attention mechanism in the graph, and the number of attention heads in it is an adjustable parameter, multiple attention heads allow the network to learn the relationship between nodes from different perspectives. To study the effect of the number of attention heads on the performance of the model, we set the number of attention heads to three, four, five, and six for the experiments, and the results are shown in Table 7, which indicates that the model is most effective when the number of attention heads is 4. Figure 3 shows the experimental results on the AVEC2019 dataset. An increase in the number of attention heads means that the model is able to capture information from different subspaces, but it also increases the number of parameters of the model. While fewer attention heads would limit the model’s ability to capture information, too many attention heads would result in a model that is too complex and prone to overfitting. When the number of heads is four, the model reaches the optimal balance between the number of parameters and the learning ability, which can effectively capture diverse feature information without overfitting due to too many parameters. In order to comprehensively understand the effect of attention heads, we conducted combination experiments with different and heads, and obtained the optimal combination of heads as 20 and 4, and the experimental results on the AVEC2019 dataset are shown in Figure 4. This combination experiment further verifies the effectiveness of the model and provides a reference for selecting the optimal attention head and context window size in different application scenarios.

### 4.4. Visualization

In this section, to better explore the performance of the comparison models, we perform several visualizations of the experiments from the previous section, including fitting the true and predicted scatterplots using regression lines, visualization of the GAT attention weights, and visualization of the model’s hidden layer representation.

#### 4.4.1. Visualization of Scatterplots

**Figure 5:**
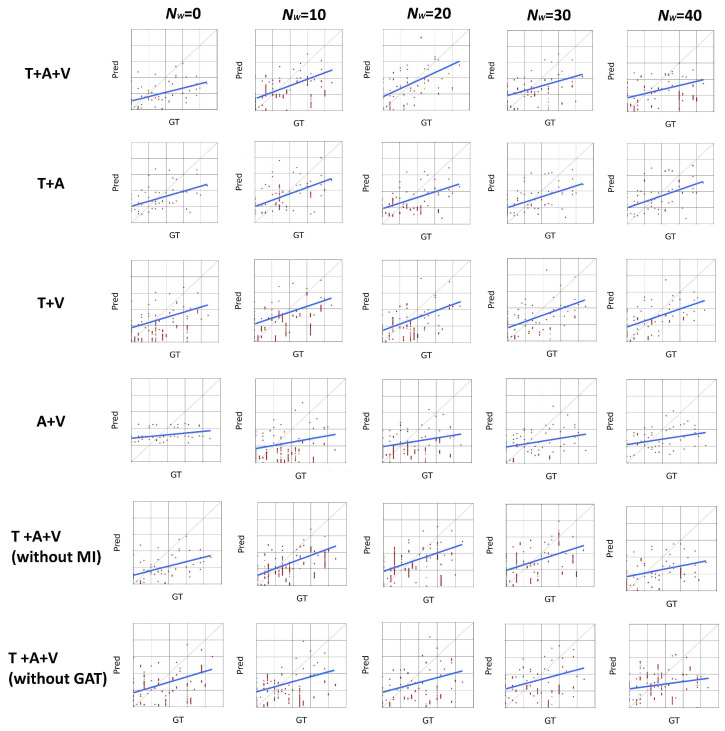
The correlation of prediction vs. ground truth on the AVEC2019 dataset under different $N_{w}$ conditions, where the basic model is T + A + V ($N_{w}$ = 20).

**Figure 6:**
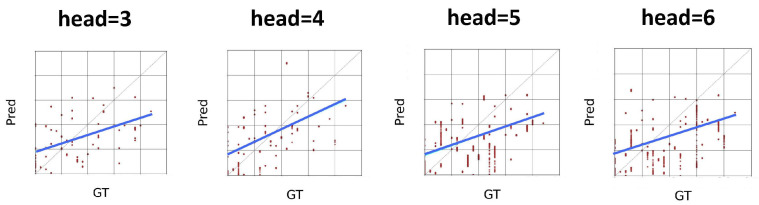
The correlation of prediction vs. ground truth on the AVEC2019 dataset with different numbers of heads.

In order to compare the predictive performance of different models, we visualize the scatter of the true and predicted values. The scatterplot goes through the distribution of data points, which reflects the correlation between the predicted and true values (it depicts how the predicted values change with the true values when they change). Ideally, these points should be distributed along a straight line with y = x (the gray dotted line in each subplot), indicating that it has a perfect prediction. If the scatterplot shows points arranged roughly along a certain straight line, but not exactly y = x, this indicates that the predicted values have some linear correlation with the true values, but there is prediction error. To better illustrate the prediction trend, we reverse-fit the scatter points into a regression line, which is highlighted in blue in each subplot. In order to investigate the effect of the context window values and the number of attention heads for different ablation modes on the model, we visualized the scatter prediction results for the predicted results separately, and the results for the context window values and the attention heads are shown in Figure 5 and Figure 6, respectively, where each row is for a different ablation model, and each column is for a different context window value and a different number of attention heads, respectively. As can be seen from the figure, the regression line between the predicted and true values is closest to 45 degrees when using the three modalities of T + A + V, $N_{w}$ = 20, and heads = 4, which indicates that this model predicts more accurately. While the regression curves of other models are more shifted from y = x curve, and their predicted value distribution is also more discrete, at the same time part of the model for a real value has a large span of predicted values, such as T + V modalities and $N_{w}$ = 10, which proves that their prediction effects are not as good as that of the model proposed in this paper.

#### 4.4.2. Visualization of Attention Weights

**Figure 7:**
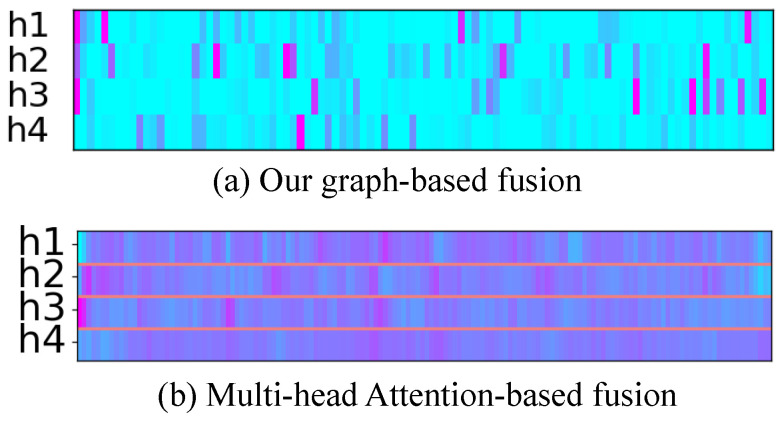
Visualization of attention weight on AVEC2019 dataset under different models. Redder color means higher weight value. The more purple the color, the higher the weight.

We visualize the attention weights of GAT and multi-head attention mechanisms to illustrate the relationships among timesteps (Figure 7). The weight graphs reveal distinct attention patterns: multi-head attention focuses on consecutive partial utterances, resulting in chunk-like attention weights, while GAT exhibits jump-like attention weights, aligning with the intermittent nature of depression symptoms (Problem 1). The sparser distribution of attention weights in GAT indicates higher model confidence, whereas the denser distribution in multi-head attention suggests lower confidence. In depression detection tasks, GAT’s ability to identify and reinforce intermittent and discontinuous associations makes it a more suitable choice. The jumping attention weights of GAT, combined with the intermittent manifestation of depression symptoms, enable effective recognition of critical but discontinuous symptom patterns. Furthermore, the GAT model’s attention weights are more scattered and adaptive, allowing it to capture complex relationships between timesteps, whereas multi-head attention tends to focus on local patterns. This adaptability makes GAT more effective at handling the variability and unpredictability of depression symptoms. Additionally, the visualization results show that GAT attention weights are more consistent across different attention heads, indicating a more robust and reliable attention mechanism. Overall, the visualization results demonstrate the superiority of GAT in capturing intermittent and discontinuous associations in depression detection tasks.

#### 4.4.3. Visualization of Hidden Layer Representations

**Figure 8:**
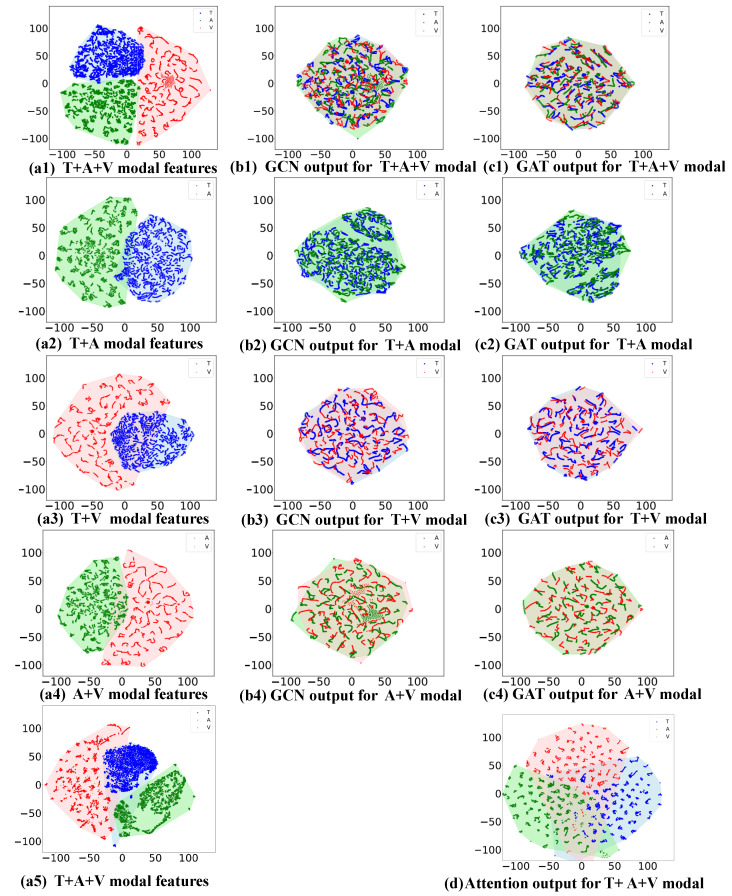
t-SNE visualization of features and graph network output of different models on AVEC2019 dataset. (**a**) Context features output by GRU; (**b**) representation output by GCN; (**c**) representation output by GAT; (**d**) representation output by multi-head attention. Red, green, and blue colors represent the audio, video, and text modalities, respectively.

We employed t-SNE dimensionality reduction to visualize the hidden layer representations of the three parts of each model in the ablation experiments. The results are shown in Figure 8. The modal features after GRU processing are depicted in Figure 8a, which corresponds to the output C in the second part of Figure 2. The figures reveal discriminative clusters, with each color representing a modal feature. The blue dots represent textual features, green dots represent audio features, and red dots represent visual features. Figure 8(a1) shows the feature visualization of the three-modal model, where the dots of each color represent the hidden layer representations of the corresponding modal features after GRU processing. The t-SNE results indicate that these dots form distinctly separated clusters in the two-dimensional space, suggesting that the features of different modalities have distinguishable spatial representations after GRU processing. This distribution implies complementarity between the different modalities in the depression detection task, where each modality provides unique and distinguishable information. Figure 8(a2–a4) show visualizations of the three bimodal models. Notably, the audio and text modal internal features exhibit relatively concentrated clustering, whereas the text–video and audio–video modal features exhibit more dispersed clustering. This is because audio and text features are processed to maintain their original structural information, whereas video features are more decentralized due to their unstructured and complex nature. This difference is attributed to the fact that video data contains richer environmental information and dynamic changes, which are more difficult to retain during dimensionality reduction. Moreover, the video modal information is more diffusely distributed in each model, indicating that the video modality is not effective for depression prediction, consistent with the results in Table 5, where a single–video modal model has the worst performance.

We also visualized the hidden layer features of different graph network modules in the depression detection model after processing multimodal data by t-SNE. The results demonstrate the difference between the GCN module alone (Figure 8b) and the GCN module followed by GAT-weighted fusion (Figure 8c). Figure 8b compares the original multimodal features through the distribution of the output features formed by the GCN-processed features in the two-dimensional space, demonstrating the GCN’s ability to capture and distinguish different modal features. However, the aggregation effect is not significant enough compared to Figure 8c. Moreover, there are many independent feature points inside each Figure 8b, indicating that the fusion effect is not good enough. As shown in Table 5, using only GCN to aggregate depression information results in unsatisfactory depression prediction performance due to its inability to dynamically adjust the importance of different neighboring nodes. In contrast, Figure 8c demonstrates the effect of the GCN output features after the second dynamic weighted fusion of GAT, which significantly enhances the differentiation and aggregation of the features, forming more compact clusters. By comparing Figure 8b and Figure 8c, we can clearly see how GAT-weighted fusion optimizes the model performance and further improves the accuracy of depression detection.

To further verify the effectiveness of the proposed model, we compared the output features of the graph fusion model with the output features of the attention mechanism fusion model (Figure 8d). The attention mechanism fusion model can only align some of the features fused, prioritizing features considered more informative. In contrast, the graph fusion model proposed in this paper shows its remarkable ability to capture overall feature alignment, providing a global feature representation by exploiting the connectivity patterns between nodes. This global perspective allows the graph network to be more comprehensive and in-depth when fusing features from different sources. Instead of considering each node in isolation, each node is evaluated in the context of the entire graph, enabling the model to capture subtle feature changes. Thus, the effectiveness of the model proposed in this paper is further validated.

## 5. Conclusions

In this work, we proposed a novel graph-based multimodal framework for automatic depression severity estimation, tailored to capture the temporally irregular nature of depressive behaviors. The model utilizes data collected via common sensors—RGB cameras and microphones—to obtain visual and acoustic signals during interviews or conversations. Unlike prior approaches that predominantly model momentary affect, our method segments long interview recordings into utterance-level instances and models their interrelations using relational and attentional graph neural networks (RGCN and GAT). This design allows the model to learn both local sequential cues and long-range symptom patterns, enabling more accurate modeling of depression’s intermittent and evolving manifestations.

Compared to the state of the art, our approach achieves substantial performance gains on two widely used benchmark datasets (AVEC2014 and AVEC2019), confirming the advantage of modeling depressive symptoms as temporally structured and crossmodally expressed signals. Our findings also suggest that a context window of around 20 utterances offers an effective granularity for estimating depressive states, potentially guiding the design of future time-aware models.

Importantly, although our framework adopts a regression-based formulation to predict continuous depression severity scores (BDI, PHQ-8), it remains clinically meaningful. These scores can be mapped to established diagnostic categories (e.g., minimal, mild, moderate, severe), facilitating integration into clinical workflows and enabling derivation of classification metrics such as sensitivity and specificity. This highlights the model’s potential for real-world screening and triaging scenarios, where early risk detection is critical.

Nonetheless, the present study has limitations. It relies on pre-collected datasets under constrained environments, and has not yet been validated in live clinical settings. The absence of interpretability mechanisms also limits its direct usability by clinicians. Future work will focus on extending the framework to larger and more diverse populations, exploring explainable modeling techniques, and embedding the model within real-time decision-making systems for mental health professionals. Such steps will be essential to bridge the gap between technical development and clinical impact.

## Figures and Tables

**Table 1:** Summary of representative multimodal depression detection models.

Methods	Backbone Network	Limitations
Early/Late Fusion [22]	Handcrafted features + fusion	Shallow feature-level fusion; lacks deep contextual representation
GRU-TriModal [7]	GRU-based fusion for text/audio/video	Does not model long-range temporal structure; limited session-level reasoning
BERT-CNN+Gated-CNN [10]	CNN + modality-specific deep networks	Independent modality modeling; lacks crossmodal alignment
MTAL [23]	Multimodal topic-enhanced model	Ignores long-term temporal progression; topic modeling not adaptive to sequences
BiGRU-Attn [24]	Text/speech encoding + attention fusion	Static fusion; lacks temporal dependency modeling
STA-MAFF [26]	Spatial–temporal attention + fusion	Focused only on local cues; lacks global session modeling
BiGRU-Attn [27]	BiGRU + attention across modalities	No modeling of intermodal structure
GRU-BiLSTM-Attn [28]	Sequential encoders + attention	No cross-utterance or session-level modeling
TensorFormer [13]	Multimodal transformer with tensor fusion	Computationally expensive; not optimized for graph structure
CAIINet [29]	Contextual attention + interaction modules	Focuses on local timepoints; lacks global coherence
HCAG [33]	Hierarchical GAT on text/audio	No utterance-level fusion; limited modality diversity
Multi-Head GAT [34]	Graph attention with intermodal fusion	Ignores chronic nature of depression; no dual-edge modeling
MS2-GNN [35]	Modality-shared/specific GNN	No full-session temporal modeling; lacks skip connections

**Table 2:** Summary of datasets used in our experiments.

Dataset	Subjects	Modality	Label Type
AVEC2014	82	Audio + Video	BDI score
AVEC2019	275	Audio + Video + Text	PHQ-8 score

**Table 3:** Summary of representative baseline models used for comparison.

Model	Description
CCA [43]	Ensemble of Canonical Correlation Analyzers for audio–visual affect prediction.
GMM+ELM [44]	Gaussian mixture models with ELM using vocal and facial features.
PLS+LR [45]	Combines partial least squares and linear regression for depression score estimation.
m-BAM [46]	Bidirectional associative memory for multisensory fusion.
MAFF [26]	Multimodal attention feature fusion framework.
AVA-DepressNet [47]	Audio–visual attention network with privacy-preserving design.
STA-DRN [48]	Spatial–temporal attention network for depression prediction.
EF [7]	Kernel-based multimodal fusion addressing cross-lingual emotion recognition.
BERT-CNN+Gated-CNN [10]	Combines BERT and CNN for multimodal fusion.
Hierarchical BiGRU [49]	BiGRU model with Focal Loss for speech emotion imbalance.
Multi-scale TDCNN [50]	Dilated CNNs over multiple time scales with BERT and statistical features.
Adaptive Fusion Transformer [51]	Transformer with adaptive fusion of multimodal signals.
DepressNet [14]	BiLSTM-based hierarchical attention model for end-to-end depression scoring.
TensorFormer [13]	Tensor-based Transformer for crossmodal interaction.
MFM-Att [52]	Multimodal fusion model capturing complex depressive phenotype.
MT [53]	Multitask model combining depression detection and sentiment analysis.
FAU-GF [54]	Uses identity-free facial muscle movement and speech cues with graph-based modeling to reduce noise and enhance episodic temporal reasoning.

**Table 4:** Comparison results of the proposed model and baselines, where (-) indicates that the relevant paper did not mention it. ↑ indicates that higher is better, ↓ indicates that lower is better; the same applies below.

**Models**	**Parameters**	**AVEC2014**	
		**MAE**↓	**RMSE**↓
CCA [43]	2.9 M	7.69	9.61
GMM+ELM [44]	3.8 M	6.31	8.12
PLS+LR [45]	2.1 M	6.14	7.43
m-BAM [46]	0.9 M	5.78	7.47
MAFF [26]	2.6 M	**5.21**	7.03
AVA-DepressNet [47]	32.1 M	5.32	6.83
STA-DRN [48]	4.7 M	6.00	7.75
**Ours**	6.1 M	5.25	**6.75**
**Models**	**Parameters**	**AVEC2019**	
		**CCC**↑	**RMSE**↓
EF [7]	0.6 M	0.344	-
BERT-CNN+Gated-CNN [10]	2.3 M	0.403	6.11
Hierarchical BiLSTM [8]	4.2 M	0.442	5.50
Multi-scale Temporal Dilated CNN [50]	0.5 M	0.430	4.39
Adaptive Fusion Transformer [51]	-	0.331	-
DepressNet [14]	7.1 M	0.457	5.36
TensorFormer [13]	65 M	0.493	**4.31**
MFM-Att [52]	26.1 M	-	5.17
MT [53]	-	0.466	-
FAU-GF [54]	15.4 M	0.555	4.95
**Ours**	6.1 M	**0.554**	4.61

**Table 5:** Comparison results of each component in the proposed model, where (-) indicates that the AVEC2014 dataset lacks the transcribed text modality.

Configurations	AVEC2014		AVEC2019	
	MAE↓	RMSE↓	CCC↑	RMSE↓
DepressionMIGNN (T)	-	-	0.531	6.21
DepressionMIGNN (A)	6.21	8.62	0.418	6.47
DepressionMIGNN (V)	7.76	7.78	0.210	7.37
DepressionMIGNN (T + A)	-	-	0.541	5.51
DepressionMIGNN (T + V)	-	-	0.522	5.44
DepressionMIGNN (A + V)	**5.25**	**6.75**	0.240	7.31
DepressionMIGNN (T + A + V)	-	-	**0.554**	**4.61**
DepressionGNN (w/o Multiple Instance)	6.41	7.43	0.458	5.64
DepressionMI (w/o GNN)	8.05	8.52	0.296	8.67
DepressionMIGCN (w/o GAT w/GCN)	6.72	7.27	0.406	6.01
DepressionMIGNN (w/o BFC)	5.96	6.33	0.425	6.12
DepressionMIGNN (w/o GNN w/Multiple Attention)	7.97	7.41	0.302	8.12

**Table 6:** Effects of different numbers of window.

Configurations	AVEC2014		AVEC2019	
	MAE↓	RMSE↓	CCC↑	RMSE↓
DepressionMIGNN ($N_{w}$ = 0)	7.38	8.07	0.324	6.68
DepressionMIGNN ($N_{w}$ = 10)	6.82	7.52	0.520	5.42
DepressionMIGNN ($N_{w}$ = 16)	6.38	7.21	0.546	5.32
DepressionMIGNN ($N_{w}$ = 20)	**5.25**	**6.75**	**0.554**	**4.61**
DepressionMIGNN ($N_{w}$ = 24)	6.06	6.98	0.375	6.19
DepressionMIGNN ($N_{w}$ = 30)	6.47	7.37	0.409	5.90
DepressionMIGNN ($N_{w}$ = 40)	6.93	8.42	0.337	6.29

**Table 7:** Effects of different numbers of heads.

Configurations	AVEC2014		AVEC2019	
	MAE↓	RMSE↓	CCC↑	RMSE↓
DepressionMIGNN (head = 3)	6.71	7.43	0.434	5.95
DepressionMIGNN (head = 4)	**5.25**	**6.75**	**0.554**	**4.61**
DepressionMIGNN (head = 5)	6.06	7.19	0.488	5.70
DepressionMIGNN (head = 6)	6.40	7.36	0.432	6.05

## Data Availability

The data presented in this study are available on request from the corresponding author.
